# The hydroxyapatite Turkish Delight: a technical note

**DOI:** 10.1007/s10006-017-0646-x

**Published:** 2017-09-01

**Authors:** L. Kustermans, Maurice Y. Mommaerts

**Affiliations:** European Face Centre, Universitair Ziekenhuis Brussel, Vrije Universiteit Brussel, Laarbeeklaan 101, B-1090 Brussels, Belgium

**Keywords:** Acquired nose deformities, Nasal surgical procedures, Autografts, Allografts, Prostheses and implants, Biocompatible materials, Hydroxyapatite, Oxidized cellulose

## Abstract

Nasal dorsum augmentation is commonly performed using autologous cartilage grafts, also in the Turkish Delight technique. The aim of this study was to describe a modification of the Turkish Delight technique for dorsal augmentation consisting of small hydroxyapatite-calcium carbonate granules (0.5–1 mm) that were wrapped in layers of oxidized cellulose and glued with 1–2-cm^3^ fibrin sealant and to compare its utility with that of other techniques. Clinically stable and satisfactory results were achieved in the four cases examined. Cone-beam computerized tomography (CBCT) imaging revealed that there was no degradation of the graft up to 2 years after surgery. The use of a modified Turkish Delight method using hydroxyapatite granules promises to be a valuable option for the correction of nasal dorsum deficiency.

## Introduction

Augmentation of the nasal dorsum has been achieved using various techniques and materials [1–3]. Autologous cartilage is most commonly used as a graft material and remains the gold standard against which other materials are compared [1]. In 2000, Erol was the first to describe the use of diced cartilage that was wrapped in a monolayer of oxidized cellulose (Surgicel®, Ethicon, Somerville, USA), termed the Turkish Delight technique [2–4]. Many modifications of this technique have been made in an attempt to improve long-term results and extend its applicability to more complicated cases [5, 7–9]. The aim of this study was to present the modified Turkish Delight method using Surgicel®-wrapped hydroxyapatite granules and compare its use with other techniques.

## Materials and methods

**Fig. 1:**
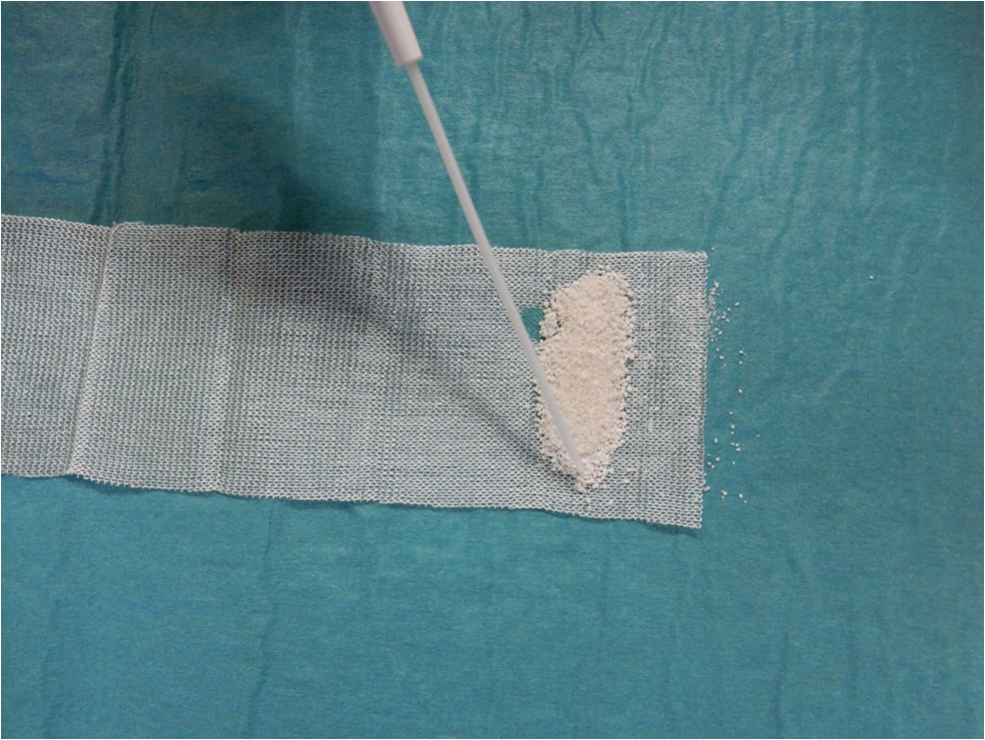
Preparing the Turkish Delight: ProOsteon® (white granules), Tisseel® (from canula), and Surgicel® (knitten fleece)

**Fig. 2:**
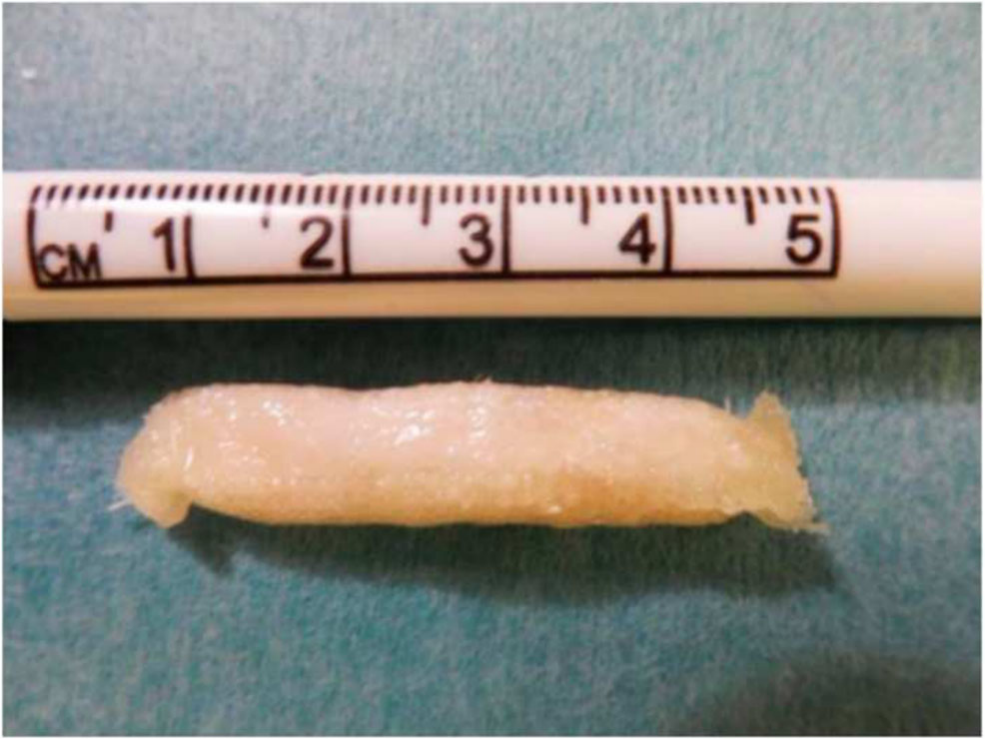
The hydroxyapatite Turkish Delight ready to implant

We used a modified Turkish Delight method using ProOsteon® 200R (Biomet, Warsaw, USA), which is a resorbable, osteoconductive, hydroxyapatite-calcium carbonate matrix, as an alloplastic graft material instead of diced autologous cartilage. Small matrix granules (0.5–1 mm) were first wrapped in layers of Surgicel® until a sausage-like shape was obtained; the Surgicel® was then moistened with 1–2 ml Tisseel® (Baxter, Zürich, Switzerland), a two-component fibrin sealant consisting of fibrinogen and thrombin (Figs. 1 and 2). A resorbable suture (Vicryl 4–0, Ethicon, Somerville, USA) was then used to tighten both ends, thereby preventing the dispersion of the granules. Finally, the wrap was soaked in rifampicin before its insertion into the recipient bed by an endonasal approach. The nasal dorsum can be molded into a more desirable shape by digital manipulation up to 3–4 weeks postoperatively. Suture strips® (Derma Sciences, Princeton, USA), and, occasionally, an external nasal Thermasplint® (Medtronic, Minneapolis, USA), were then applied to preserve the shape of the graft.

## Results

**Fig. 3:**
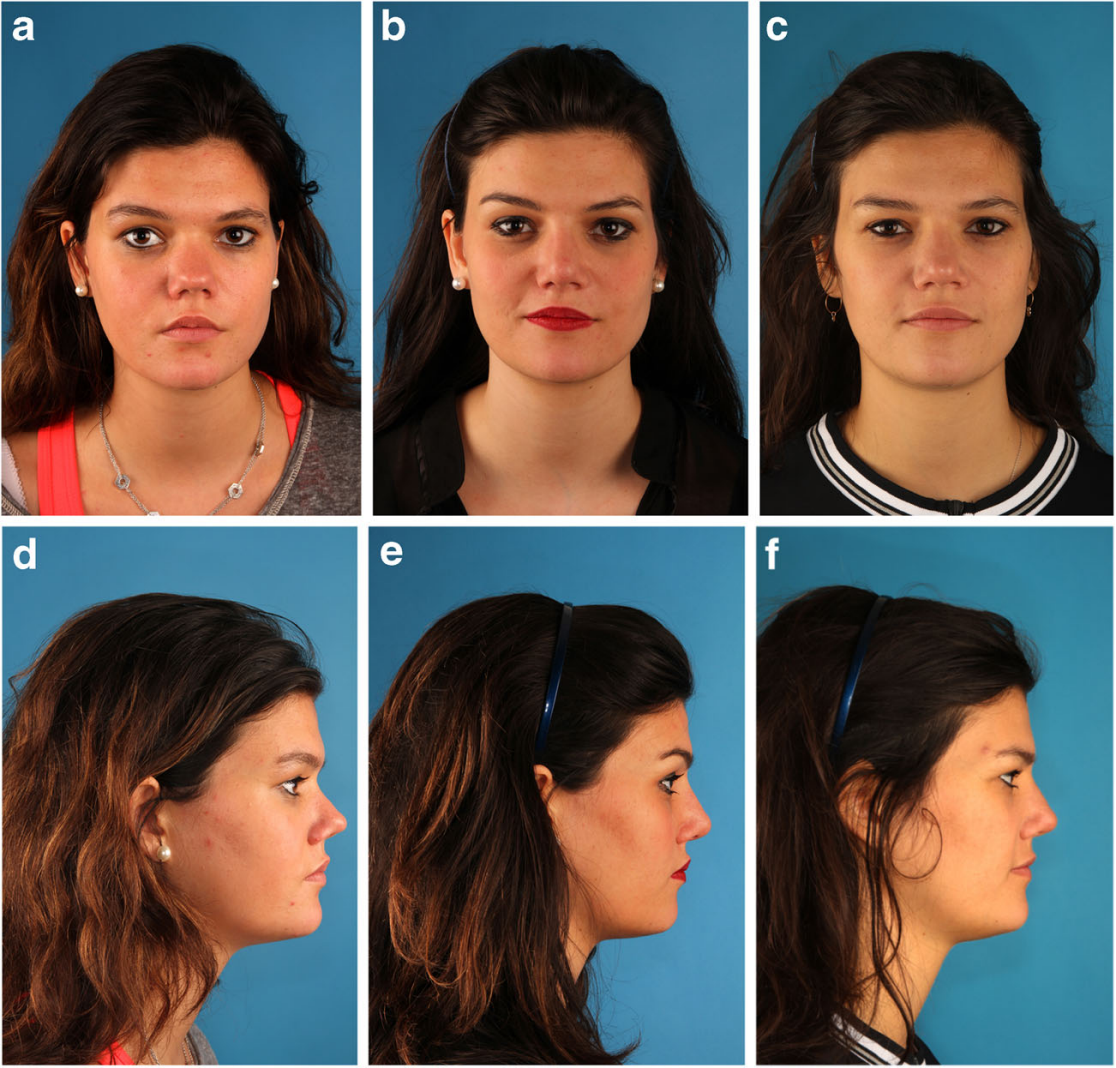
Preoperative and postoperative photographs. **a** Preoperative face frontal view. **b** One-month postoperative face frontal view. **c** Two-year postoperative face frontal view. **d** Preoperative face profile view. **e** One-month postoperative face profile view. **f** Two-year postoperative face profile view

**Fig. 4:**
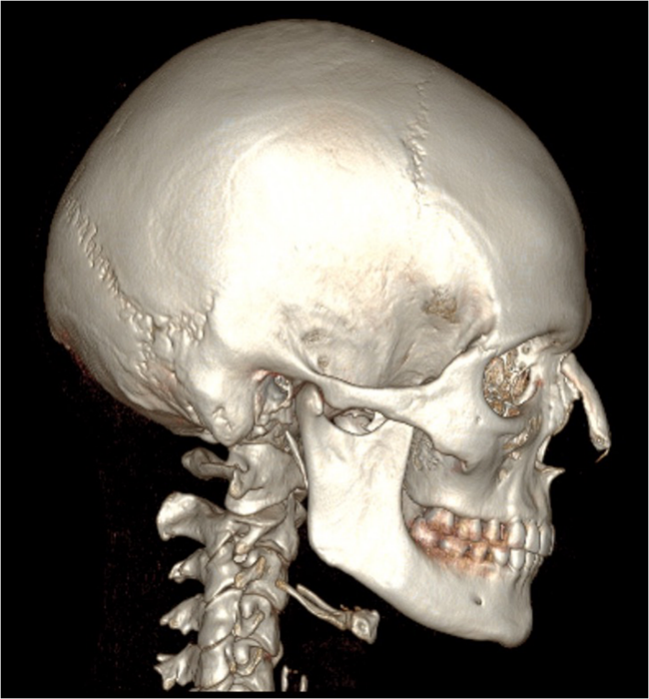
CBCT scan 2 years postoperatively of the patient in Fig. 3

**Fig. 5:**
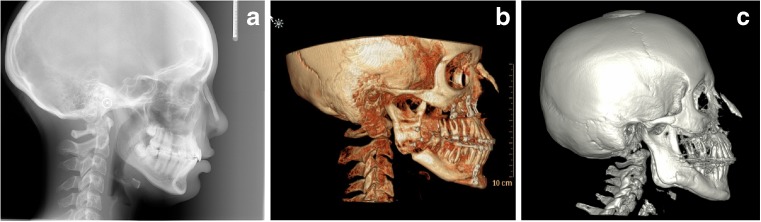
Postoperative radiographic imaging of the other three cases. **a** Four-month postoperative lateral cephalogram. **b** Six-month postoperative CBCT scan. **c** One-year postoperative CBCT scan

We found this modified Turkish Delight method to be a reliable technique for the correction of nasal dorsum deficiencies. A total of four patients with a structural deficiency of congenital etiology, including one patient suffering from a unilateral cleft lip, were treated in this manner and followed for 4 months to 2 years. All patients observed were women between the ages of 17 and 32 years without significant previous medical history. Although no quantitative measurements were performed in our small series, clinically stable and satisfactory results were achieved (Fig. 3). CBCT images, taken up to 2 years after surgery, support our clinical findings of stability (Fig. 4). Moreover, they show a complete mineralization of the Turkish Delight over time, with discrete hydroxyapatite granules still apparent at 4 months postoperative, to become a homogenous sintered mass 1 year after (Fig. 5).

## Discussion

Cartilage, bone, fascia, dermis, fat, and synthetic products have been assessed for their use in nasal dorsum grafting. A minimal inflammatory response is induced by autologous cartilage, with low graft infection, resorption, and extrusion rates. Irradiated homologous cartilage grafts induce resorption in 1–7.4% of cases, displacement in 0.3–5.9% of cases, and warping in 1–14.8% of cases [1]. The use of silicone and polyethylene results in significant rates of infection (up to 4%), displacement, extrusion (up to 10%), and exposure due to chronic inflammation with encapsulation and lack of biointegration [1].

The nasal cartilaginous septum is the most frequently used graft material for limited augmentation, mainly because it obviates the need for an extra donor site [1]. This material can be reshaped, carved, diced, or crushed to match the defect. However, its availability is limited, particularly in revision rhinoplasty if the septal cartilage has been harvested previously. Drawbacks in the use of autologous grafts from a distant site, especially rib grafts, include prolonged surgical time and complexity, donor-site morbidity, difficult graft shaping, graft displacement (1–12.5%) [1], and an unpredictable rate of resorption (0.5–5%) [3, 4]. With solid cartilage grafts, the bone-cartilage transition can be viewed through the nasal skin after the resolution of edema [3, 4].

In 2000, Erol [2] described the use of autogenous septal cartilage that was diced into small (0.5–1-mm) pieces, infused with 1–2 ml of the patient’s blood, wrapped in a monolayer of oxidized cellulose (Surgicel®), and moistened with an antibiotic (rifamycin). This technique is referred to as Turkish Delight [3, 4]. Wrapping the material prohibits the dispersion of the cartilaginous pieces into the recipient’s tissues and allows the nasal dorsum to be reconstructed using digital pressure. Microscopically, a mosaic-type fibro-cartilaginous alignment can be observed, which produces a smooth surface and discourages late visibility. Erol’s original series of 2365 patients, who were followed for 4–25 years, shows stable, long-term results; while both partial and excessive resorption were observed, the reported undercorrection was only 0.4 and 0.05% of his patients, respectively [4]. This technique became rapidly popular but a significant rate of graft resorption within a few months after implantation of up to 30% (clinically) and 69% (experimentally) of the original thickness was reported by others [3]. Some researchers have speculated that inflammation and impaired revascularization from the Surgicel® wrap are responsible for these high rates of resorption, as fibrosis, lymphocytic infiltrates, and metabolically inactive remnants of cartilage were all observed [3]. Bayram et al. used slightly to moderately crushed cartilage beneath a monolayer of Surgicel® and observed a low resorption rate of 2.4% [6], whereas other researchers have reported rates between 12 and 50%, depending on the degree of material crushing involved. Cartilage that is crushed or fragmented, and then wrapped in fascia instead of Surgicel®, results in less resorption due to the survival of cartilage cells with normal metabolic activity. Donor-site morbidity in the temporal region remains the major drawback of retrieving a fascial graft to obtain a suitable nasal contour and/or to correct deficiencies of the nasal dorsum exceeding 2 mm; thus, fascial grafts should only be harvested in those patients with a thin skin [5]. Richardson et al. [7] replaced the cartilaginous graft material with finely diced Medpor® (expanded polyethylene; Porex Surgical Inc., Newnan, USA) mixed with 1–2 cm^3^ of the patient’s blood, whereas Bullocks et al. tried to stabilize the graft structure by the addition of platelet-rich plasma and platelet-poor plasma instead of wrapping [8]. Although this method was time-consuming, significantly less resorption was observed by minimizing the degree of inflammation and permitting better revascularization. Similarly, Tasman et al. [9] developed a “glue graft,” consisting of diced cartilage impregnated with a fibrin sealant; these authors did not observe any resorption, besides of the fibrin glue itself, during the early postoperative phase (mean of −3% after 4–9 months). However, while the fragility of the structure necessitated the preparation of a wide surgical access to the recipient bed, the use of an endonasal approach was still feasible according to the authors***.***

Using readily available hydroxyapatite granules (ProOsteon), a fibrin sealant, and the Surgicel® wrap, the advantages of some of the above-mentioned techniques can be used. The porosity of the granules allows fibro-vascular ingrowth to enhance stabilization and vascularization of the implant. ProOsteon does not require special handling or reconstitution, and is shipped sterile and ready-to-use. A second surgical site can be avoided by respecting the algorithm proposed by Bohluli et al., who suggested the use of easier and more conservative techniques first before considering secondary donor sites in cases where no suitable result can be achieved [6]. The manufacturer (Biomet) currently states that ProOsteon degrades after approximately 6 months [10]. However, there are no conclusive data regarding the long-term replacement of large graft volumes [11]. For craniofacial purposes, it is advantageous to use a material that undergoes little or no degradation. A published study reported the long-term stability of this material, since the cranio-maxillofacial area does not undergo significant weight-bearing loads [12]. Additionally, Pollick et al. observed a fast rate of degradation when the graft material was deposited in bony defects but not in soft tissues. Within 4 months, the resorption rate was 24–63% in bone but was not statistically significant in either intramuscular or subcutaneous tissues [13]. Furthermore, radiographic results of esthetic augmentation of the facial skeleton using porous hydroxyapatite granules in 10 female patients demonstrated that full bony projections (99.7%) were maintained after 2 years [11]. Such studies support our belief that the modified Turkish Delight method using hydroxyapatite granules is a reliable technique for nasal dorsum augmentation.
